# Fertility awareness among medical and non-medical students: a case-control study

**DOI:** 10.1186/1477-7827-12-94

**Published:** 2014-09-26

**Authors:** Kazem Nouri, Dagmar Huber, Katharina Walch, Regina Promberger, Bernd Buerkle, Johannes Ott, Clemens B Tempfer

**Affiliations:** Department of Obstetrics and Gynecology, Medical University of Vienna, Waehringer Guertel 18-20, 1090 Vienna, Austria; Clinical Department of Gynecologic Endocrinology and Reproductive Medicine, Medical University of Vienna, Waehringer Guertel 18-20, 1090 Vienna, Austria; Department of Surgery, Medical University of Vienna, Waehringer Guertel 18-20, 1090 Vienna, Austria; Department of Obstetrics and Gynecology, Ruhr University Bochum, Bochum, Germany

**Keywords:** Fertility, Awareness, Questionnaire, Medical students, Family planning

## Abstract

**Background:**

To compare the understanding and perceptions of fertility issues among medical and non-medical University students.

**Methods:**

In a prospective case-control study, using a 43 item questionnaire with 5 sections and 43 questions regarding personal data (8 questions), lifestyle factors (9 questions), plans on having children (5 questions), age and fertility (5 questions), and lifestyle and fertility (16 questions), knowledge of fertility and influencing factors, desired age at commencement and completion of childbearing, among male and female medical and non-medical students in their first academic year at Vienna University, Vienna, Austria were evaluated.

**Results:**

340 students were included. 262/340 (77%) participants planned to have children in the future. Medical students (n = 170) planned to have fewer and later children and had a higher awareness of the impact of age on fertility than non-medical students (n = 170; estimated knowledge probability 0.55 [medical students] vs. 0.47 [non-medical students]; F (1, 336) = 5.18 and p = .024 (η p = .015). Gender did not independently affect estimated knowledge probability (F (1, 336) = 1.50 and p = .221). More female and male medical students had a positive attitude towards Assisted Reproductive Technology in case of infertility than non-medical students (47 and 55% vs. 23 and 29%, respectively; p = <.001). Medical students had a healthier lifestyle than non-medical students. A healthy lifestyle and female gender were associated with higher fertility awareness.

**Conclusions:**

Medical students have a higher awareness of fertility issues than non-medical students. Choice of academic study, gender, and personal life style are important factors affecting fertility awareness. These data may be helpful to address knowledge gaps among young non-medical Academics.

**Electronic supplementary material:**

The online version of this article (doi:10.1186/1477-7827-12-94) contains supplementary material, which is available to authorized users.

## Background

A general trend towards postponing childbirth has been described in Western industrialized countries [1, 2]. The age of women at the time of their first delivery is constantly increasing, especially among women with higher education. In Austria, for example, the mean age of first-time mothers has been constantly rising over the last 20 years and is now at 28.8 years [3]. The same development can be observed in other industrialized countries, underlining the fact that many couples in affluent societies postpone their family planning until ages when female fertility has already started to decrease [4, 5]. When comparing Austrian data with those of other European countries, the age of Austrian first-time mothers lies in the middle of the spectrum, as does the age of women at the birth of their first child in the United Kingdom (mean age 27.8 years in 2010), the Czech Republic (mean age 27.8 years in 2011 compared to 22.4 years in 1990), Croatia (mean age 27.9 years in 2011 compared to 25.0 years in 1995), and Norway (mean age 28.5 years in 2012 compared to 25.5 years in 1990). The highest and lowest mean ages at first-time motherhood have been reported for Italy (mean age 30.3 years in 2011 compared to 26.9 years in 1990) and Estonia (mean age 26.4 years in 2011 compared to 22.7 years in 1990) [6]. This general trend has a significant impact on fertility and pregnancy outcome. Fecundity and fertility decrease with age whereas fetal and maternal complications associated with pregnancy and delivery progressively increase with age [7]. For example, there is a decrease in a woman’s chance of spontaneous conception in her late 20s with a marked decrease of fecundity between 35 and 39 years. Women >35 years can expect a 2-fold longer time to conception compared to women aged <25 years [8, 9]. Similar to what has been observed among women, there is also a trend among men to have their first children at an older age [10]. Although the effects of age on fecundity and fertility are much more pronounced in women, fertility also significantly declines with increasing age in men. There is a detectable decline in semen parameters after the age of 35 and an appreciable decrease in male fertility beyond the age of 50 years [11, 12]. Besides age, many other factors affect fertility in both men and women, among them life-style, diet, exercise, and obesity [13–16].

A number of studies have been conducted assessing fertility awareness among young men and women. In summary, fertility awareness in general and the knowledge of specific factors influencing fertility in particular have been described to be unrealistic among many young couples [17–19]. This is also true for University students. For example, Tyden et al. came to the conclusion that female University students are not very concerned about having children before they get ‘too old’ [20]. An Italian study showed that although students attribute a noticeable importance to parenthood, their knowledge about human reproduction is surprisingly poor [4]. In a survey of Swedish University students in 2004, Lampic et al. found that most of the participants wanted to become parents at some point in their lives and had a realistic perception of the most fertile period in a woman’s life. However, specific issues such as female fecundity were markedly overestimated [5]. Among Finnish University students, over half of the men and one-third of the women believed that the age when a marked decrease in female fertility begins is over 45 years [21]. In accordance with these data, Hammarberg et al. found that the majority of surveyed couples underestimated the initiation of the natural decrease in fertility by about 10 years [7]. Based on a literature search (PUBMED search; search date: 20-06-2014; search terms: fertility awareness, survey, questionnaire, gender, students), little is known about gender-specific differences in fertility awareness and variations among groups of students such as medical and non-medical students. Therefore, we investigated the attitudes, knowledge and awareness of female and male medical and non-medical students regarding parenthood and fertility issues using a 43 item questionnaire in a prospective case-control study setting.

## Methods

The present study was approved by the Ethics Committee of the Medical University of Vienna (registration number 1282/2012). The study was designed as a prospective case-control, questionnaire-based investigation including first-year medical and non-medical University students. The questionnaire was handed out among students of the Medical University of Vienna, the University of Vienna, and the Vienna University School of Economics and Business between October and November 2012. Students from other faculties were excluded. Participants were randomly selected after a formal class. Inclusion criteria were (i) being enrolled as a Medical or non-Science student, (ii) being in the first academic year, and (iii) age between 18 and 25 years. After informed consent, students were asked to individually and anonymously fill out the questionnaire and hand it back. The study population was balanced in a 1:1 manner between medical and non-medical and between male and female students.

### Questionnaire

The anonymous questionnaire was designed by a team of two Reproductive Medicine specialists, a Gynecologic Endocrinologist and a Medical student at the Clinical Department of Gynecologic Endocrinology and Reproductive Medicine, Department of Obstetrics and Gynecology, Medical University of Vienna, Vienna, Austria. Some questions were adopted from questionnaires previously published in similar projects in other countries [4, 5, 17–21]. In June 2012, a validation study was carried out and the questionnaire was tested in each study group (medical students: n = 15 and non-medical students: n = 15) in order to test the instrument’s validity and reliability. The questionnaire was then revised on the basis of the observations made by the investigators as well as comments of the surveyed participants. The final questionnaire consisted of 43 questions and was divided into the following 5 sections: personal data (8 questions), lifestyle factors (9 questions), plans on having children (5 questions), age and fertility (5 questions), and lifestyle and fertility (16 questions). The questions had a multiple-choice design with 3 to 6 possible answers, one of which was correct. Participants were asked to choose one answer, which seemed to be most likely correct. The questionnaires were handed out among the students right after a formal lecture. The students were asked to answer to the questions individually and anonymously. The number of participants (n) was 30 for the pilot project, and 340 for the study. Further 22 questionnaires were handed out, but weren’t returned to the researcher. Thus, the response rate was 93.92%.

### Statistical analysis

Students were grouped according to type of curriculum (medicine versus non-science) and gender (male versus female) and analyzed by means of descriptive and interferential statistics. The statistical methods used in this study were cross tabulations and chi-square tests. In cases where the expected value was <5 and >20% of the cells are affected, Fisher’s exact test was used for correction. For relationships between variables, Spearman’s rank correlations were used. In order to test the difference in knowledge about the impact of age on fertility in dependence of gender and type of curriculum, a two-way ANOVA test was used. Analysis of variance was assessed in a general linear model. Interactions between variables were tested by two-way ANOVA. If the test for interaction showed a significant result, a super-additive effect of the combination of the variables was presumed. The level of significance was p < 0.05. The analysis of the data was conducted via IBM SPSS® Statistics 21 and Microsoft Excel.

## Results

In total, 340 students were included in the study. The study population was balanced between male (n = 170) and female students (n = 170) as well as medical (n = 170) and non-medical students (n = 170). Non-medical students were enrolled in the following curricula: Politics (n = 53), Economics (n = 33), Law (n = 19), Languages (n = 17), History (n = 10), Journalism (n = 9), Psychology (n = 7), International Development (n = 6), Philosophy (n = 5), Art History (n = 3), Drama (n = 3), Social Sciences (n = 2), Communication Studies (n = 1), Sports (n = 1), and Educational Sciences (n = 1). The mean age of the study population was 20.03 (±1.77) years. 256 (75.3%) of the participants were single and 84 (24.7%) were in a relationship. None of the participants were married or divorced. 4/340 (1%) students already had children.

**Figure 1:**
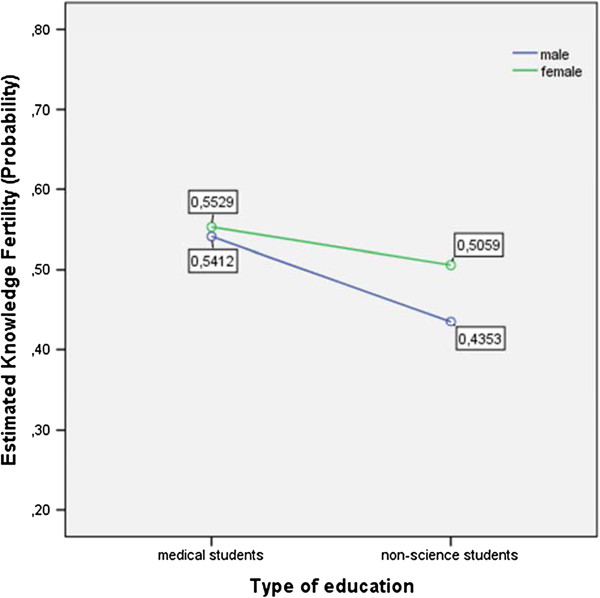
**Means of males’ and females’ knowledge of the impact of age on female fertility (estimated knowledge probability) in dependence of gender and study curriculum.**

**Figure 2:**
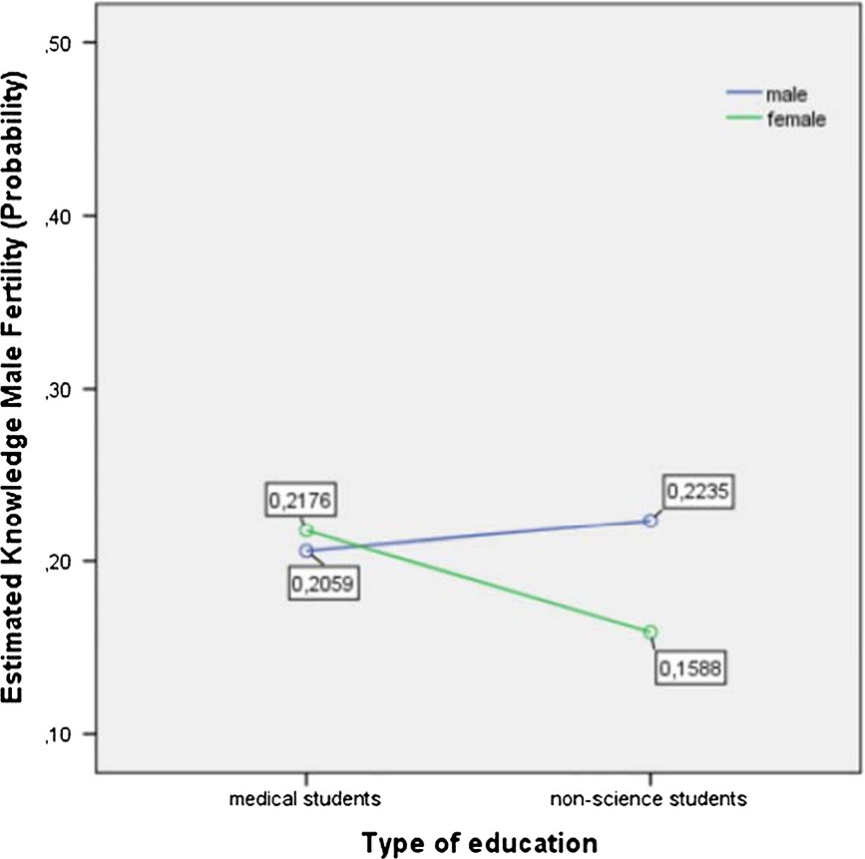
**Means of males’ and females’ knowledge of the impact of age on male fertility (estimated knowledge probability) in dependence of gender and study curriculum.**

Additional file 1 gives an example of the 43 item questionnaire used in this study. Tables 1, 2 and 3 show the questions and answers of the questionnaire’s 5 categories regarding personal data (8 questions), lifestyle factors (9 questions), plans on having children (5 questions), age and fertility (5 questions), and lifestyle and fertility (16 questions), broken down by gender and type of curriculum. Figure 1 shows the means of males’ and females’ knowledge of the impact of age on female fertility as estimated knowledge probability, broken down by gender and study curriculum. Figure 2 shows the means of males’ and females’ knowledge of the impact of age on male fertility as estimated knowledge probability, broken down by gender and study curriculum.

**Table 1:** **Questions and answers regarding the study participants’ plans on having children (5 questions) broken down by gender and type of curriculum**

Question	Answer	Gender and type of curriculum (%)				P-value
		Female non-medical	Female medical	Male non-medical	Male medical	
1. Do you want to have children (have more children) at some point in your life?	Yes	74.1	81.2	74.1	78.8	NS
	No	5.9	5.9	9.4	5.9	
	Don’t know	20.0	12.9	16.5	15.3	
2. How many children would you like to have?	1	7.5	3.8	2.7	3.8	NS
	2	42.5	66.3	40	48.8	
	3	26.3	13.8	20	22.5	
	4	1.3	2.5	4.0	2.5	
	5	0	1.3	0	1.3	
	>5	1.3	0	0	0	
	Don’t know	21.3	12.5	33.3	21.3	
3. At what age would you like to have your first child?	<20	1.3	0	0	0	NS
	20-24	5.0	1.3	1.4	1.3	
	25-29	53.8	46.3	35.6	30.0	
	30-34	30.0	37.5	45.2	52.5	
	35-39	1.3	2.5	5.5	3.8	
	40-44	0	1.3	0	0	
	≥45	0	2.5	0	0	
	Don’t know	8.8	8.8	12.3	12.5	
4. At what age would you like to have your last child?	<20	0	0	0	0	.002
	20-24	0	0	0	0	
	25-29	8.8	0	4.0	2.5	
	30-34	38.8	46.9	24.0	23.8	
	35-39	25.0	31.7	34.7	35.0	
	40-44	5.0	1.3	12.0	16.3	
	≥45	0	2.5	1.3	0	
	Don’t know	22.5	17.7	24.0	22.5	
5. In case of infertility, which option would you most likely choose?	ART	23.1	47.4	28.8	55.1	<.001
	Adoption	71.8	52.6	53.4	35.9	
	Abstain from children	5.1	0	17.8	9.0

**Table 2:** **Questions and answers regarding age and fertility (5 questions) broken down by gender and type of curriculum**

Question	Answer	Gender and type of curriculum (%)				P-value
		Female non-medical	Female medical	Male non-medical	Male medical	
1. At what age are women most fertile?	Correct	82.4	89.4	70.6	87.1	.006
	False	17.6	10.6	29.4	12.9	
2. At what age is the first decrease of female fertility?	Correct	51.8	55.3	42.4	62.4	NS
	False	48.2	44.7	57.6	37.6	
3. At what age is the second decrease of female fertility?	Correct	17.7	21.2	17.7	12.9	NS
	False	82.3	78.8	82.3	87.1	
4. At what age is the first decrease of male fertility?	Correct	17.7	25.9	18.8	18.8	NS
	False	82.3	74.1	81.2	81.2	
5. At what age is the second decrease of male fertility?	Correct	14.1	17.7	25.9	22.3	NS
	False	85.9	82.3	74.1	77.7

**Table 3:** **Questions and answers regarding life-style and fertility (16 questions) broken down by gender and type of curriculum**

Question	Answer	Gender and type of curriculum (%)				P-value
In my opinion,…		Female non-medical	Female medical	Male non-medical	Male medical	
…the consumption of **caffeinated beverages** affects female fertility in the following way:	Increases female fertility	1.2	0	3.6	0	.03
	Doesn’t affect female fertility	30.6	23.5	19.3	34.1	
	Regular consumption reduces female fertility, occasional consumption doesn’t	57.7	71.8	61.5	60.0	
	Affect it reduces female fertility	10.6	4.7	15.7	5.9	
…**alcohol consumption** affects female fertility in the following way:	Increases female fertility	2.4	0	1.2	2.4	NS
	Doesn’t affect female fertility	9.4	3.5	3.5	8.2	
	Regular consumption reduces female fertility, occasional consumption doesn’t	54.1	64.7	52.9	60.0	
	Affect itreduces female fertility	34.1	31.8	42.4	29.4	
…**smoking** affects female fertility in the following way:	Increases female fertility	3.5	1.2	2.4	0	NS
	Doesn’t affect female fertility	8.2	5.9	3.5	4.7	
	Heavy smoking reduces female fertility, occasional smoking doesn’t affect it	29.4	35.3	36.5	17.7	
	Reduces female fertility	58.8	57.7	57.7	77.7	
…**moderate exercise** affects female fertility in the following way:	Increases female fertility	48.2	55.3	60.0	76.5	.001
	Doesn’t affect female fertility	45.9	43.5	40.0	23.5	
	Reduces female fertility	5.9	1.2	0	0	
…**intense exercise** affects female fertility in the following way:	Increases female fertility	24.7	27.1	28.2	28.6	NS
	Doesn’t affect female fertility	35.3	24.7	25.9	17.9	
	Reduces female fertility	40.0	48.2	45.9	53.6	
…**excess weight** affects female fertility in the following way:	Increases female fertility	4.7	2.4	4.7	3.6	NS
	Doesn’t affect female fertility	38.8	25.9	36.5	29.8	
	Reduces female fertility	56.5	71.8	58.8	66.7	
…**underweight** affects female fertility in the following way:	Increases female fertility	2.4	1.2	1.2	0	.009
	Doesn’t affect female fertility	9.4	7.1	23.5	8.2	
	Reduces female fertility	88.2	91.8	75.3	91.8	
…a **healthy, balanced diet** affects female fertility in the following way:	Increases female fertility	83.5	86.9	87.1	89.1	NS
	Doesn’t affect female fertility	11.8	11.9	12.9	9.4	
	Reduces female fertility	4.7	1.2	0	1.2	
…the consumption of **caffeinated beverages** affects male fertility in the following way:	Increases male fertility	3.5	1.2	7.1	2.4	.007
	Doesn’t affect male fertility	35.3	27.1	17.7	32.9	
	Regular consumption reduces male fertility, occasional consumption doesn’t affect it	47.1	67.1	55.3	54.1	
	Reduces male fertility	14.1	4.7	20.0	10.6	
…**alcohol consumption** affects male fertility in the following way:	Increases male fertility	4.7	0	0	0	.03
	Doesn’t affect male fertility	5.9	2.4	5.9	10.6	
	Regular consumption reduces male fertility, occasional consumption doesn’t affect it	52.9	61.2	47.1	58.8	
	Reduces male fertility	36.5	36.5	47.1	30.6	
…**smoking** affects male fertility in the following way:	Increases male fertility	3.5	1.2	2.4	0	NS
	Doesn’t affect male fertility	8.2	5.9	4.7	7.1	
	Heavy smoking reduces male fertility, occasional smoking doesn’t affect it	29.4	31.8	30.6	17.7	
	Reduces male fertility	58.8	61.2	62.4	75.3	
…**moderate exercise** affects male fertility in the following way:	Increases male fertility	49.4	49.4	74.1	76.2	<.001
	Doesn’t affect male fertility	47.1	48.2	23.5	23.8	
	Reduces male fertility	3.5	2.4	2.4	0	
…**intense exercise** affects male fertility in the following way:	Increases male fertility	27.1	32.9	43.5	40.5	NS
	Doesn’t affect male fertility	41.2	29.4	29.4	28.5	
	Reduces male fertility	31.7	37.7	27.1	31.0	
…**excess weight** affects male fertility in the following way:	Increases male fertility	1.2	1.2	1.2	0	NS
	Doesn’t affect male fertility	38.8	23.5	30.6	27.1	
	Reduces male fertility	60.0	75.3	68.2	72.9	
…**underweight** affects male fertility in the following way:	Increases male fertility	1.2	1.2	2.4	0	.01
	Doesn’t affect male fertility	28.2	10.6	25.8	29.4	
	Reduces male fertility	70.6	88.2	71.8	70.6	
…a **healthy, balanced diet** affects male fertility in the following way:	Increases male fertility	84.7	85.9	87.1	87.1	NS
	Doesn’t affect male fertility	11.8	12.9	12.9	12.9	
	Reduces male fertility	3.5	1.2	0	0

### Plans on having children, age at first child

The majority of students (262/340 [77%]) planned to have children in the future and there was no significant difference between medical and non-medical students (Table 1). Female as well as male medical students wanted to have fewer children at a later stage in life compared to non-medical students. For example, 66% of female medical students planned to have 2 children compared to 42% of female non-medical students, whereas 3 children were an option for only 13% compared to 26%, respectively (Table 1). More non-medical than medical students preferred a low age (<29 years) at the time of having the first child (60 versus 47% for females and 37 versus 31% for males, respectively). The percentage of participants who planned on having only one child was generally low (from 2.7% [male non-medical students] to 7.5% [female non-medical students]), as was the percentage of participants, who planned on having ≥4 children (from 2.6% [female non-medical students] to 4.0% [male non-medical students]).

### Reaction to infertility, age and infertility

More female and male medical students had a positive attitude towards Assisted Reproductive Technology in case of infertility than non-medical students (47 and 55% vs. 23 and 29%, respectively; p = <.001). In addition, the option of abstaining from having children was chosen by no female medical student (0%), whereas female non-medical students chose this option in 5% (Table 1). In accordance, adoption was a realistic answer to infertility for only 53% of female medical students compared to 72% of female non-medical students.

Medical students had a higher awareness of the impact of age on female fertility than non-medical students. The estimated knowledge probability was 0.55 for medical students vs. 0.47 for non-medical students. This difference was statistically significant with F (1, 336) = 5.18 and p = .024 (η p = .015) (Figure 1). However, the calculation of the test statistic for the effect of gender on estimated knowledge probability did not show a significance with F (1, 336) = 1.50 and p = .221. In contrast, the awareness of the impact of age on male fertility was well established among female and male medical students (estimated knowledge probability 0.21 and 0.20, respectively) as well as male non-medical students (estimated knowledge probability 0.22), whereas female non-medical students reached the lowest marks (estimated knowledge probability 0.15) (Figure 2). Table 1 summarizes the questions and answers about age and fertility, broken down by gender and type of curriculum. There was a significant difference (p = .006) in the knowledge of a woman’s most fertile age between the four groups of participants with the highest probability of a correct answer among female medical students (89.4%) and the lowest probability among male non-medical students (70.6%). Female medical students reached a higher percentage of correct answers in all questions compared to female non-medical students, whereas this difference was less pronounced in males (Table 1).

### Lifestyle and infertility

Medical students had a higher awareness of the impact of lifestyle on fertility than non-medical students. Female medical students in particular reached a higher percentage of correct answers compared to female non-medical students, whereas this difference was less pronounced in males (Table 3). Compared to female non-medical students, female medical students reached a higher percentage of correct answers regarding female and male fertility in 15/16 questions. For example, questions regarding the influence of caffeine, alcohol, smoking, moderate/intense exercise, excess weight/underweight, and diet on female fertility were answered correctly by 72%, 65%, 35%, 55/48%, 72/92%, and 87% of female medical students compared to 58%, 54%, 29%, 48/40%, 56/88%, and 84% of female non-medical students, respectively. Also, the percentage of correct answers regarding male fertility was higher among female medical students compared to female non-medical students (Table 3). On the other hand, this difference was not evident when comparing male medical and non-medical students. Among male medical students, the percentage of correct answers regarding female and male fertility was higher in only 10/16 questions compared to male non-medical students.

### Lifestyle among participants

Medical students had a healthier lifestyle than non-medical students. This difference was true for both female and male medical students. Specifically, the rates of regular consumption of caffeine as well as the rate of regular smokers was lower among female medical students compared to female non-medical students (77% and 9% versus 81% and 20%, respectively; p < .05) and male medical students compared to male non-medical students (80% and 6% versus 86% and 40%, respectively; p < .05). Regular consumption of alcohol (moderate to high), however, was equally distributed among female medical students and female non-medical students (34% and 32%, respectively; p = n.s.), whereas male medical students reported significantly less alcohol consumption compared to male non-medical students (48% and 76%, respectively; p < .05). Also, regular exercise and a positive attitude towards a healthy diet were more popular among medical students (47% and 67% for female and male medical students versus 38% and 56% for female and male non-medical students, respectively; p < .05 and p < .05, respectively).

In addition, the body mass index (BMI) of the study probands was strongly associated with their awareness regarding the negative impact of excess weight on female fertility (p = .003). The lower the BMI, the higher was the awareness. This association was also observed for the probands’ own exercise habits and their awareness of a positive impact of exercise on male fertility (p = .006). The more probands exercised themselves, the more they believed in a positive effect of exercise on male fertility. Lastly, there was a significant association between the probands’ smoking and caffeine consumption habits and their awareness regarding the negative impact of smoking and caffeine on female fertility (p = .01 and p = .001, respectively). The more probands smoked and consumed caffeine, the more negligent they became.

## Discussion

In this questionnaire-based case-control study, we assessed differences in the understanding and perceptions of fertility issues among medical and non-medical University students. Using a 43 item questionnaire, we found that medical students had a higher awareness of fertility issues than non-medical students, but still tended to postpone their family planning and wanted to have fewer children. Also, medical students had a healthier lifestyle and a more positive attitude towards Assisted Reproductive Technology in case of infertility compared to non-medical students. Among all probands, a healthy lifestyle was associated with higher fertility awareness.

Our data are new regarding the impact of study curriculum and gender on fertility awareness and personal lifestyle. Based on a literature search (PUBMED search; search date: 20-06-2014; search terms: fertility awareness, survey, questionnaire, gender, students), little is known about gender-specific differences in fertility awareness and variations among groups of students such as medical and non-medical students. The results of our investigation add to the literature assessing fertility awareness among young Academics. In contrast to previous studies among University students demonstrating a general lack of interest in and knowledge of specific fertility issues [4, 5, 21], our study found that both female and male medical students had a high level of fertility awareness. We can, however, confirm that fertility awareness in general and the knowledge of specific factors influencing fertility in particular are unrealistic among many young Academics, especially male non-medical students. Also, non-medical students consume more caffeine and alcohol, are heavier smokers and less motivated exercisers than their medical counterparts. Moreover, they underestimate the impact of age on fertility and overestimate the length of the female reproductive period, as demonstrated in previous studies [5, 7, 21]. Therefore, our data suggest that there is a need for fertility education among non-medical students, which is currently not met.

Another interesting issue is the influence of gender on fertility awareness and lifestyle. Our data clearly show that male medical students and even more so male non-medical students consume more alcohol and caffeine, have a higher rate of regular smokers, and exercise less than their female counterparts. Also, the knowledge of specific fertility issues was more profound among females than males with female medical students scoring the highest results. These data indicate that fertility-awareness is a gender-specific question. Consequently, efforts to improve knowledge about fertility should target young males and refer to their knowledge gaps as outlined in this study. Better knowledge about factors negatively affecting fertility is also a societal priority, because delaying childbearing based on incorrect perceptions of female fertility may increase the burden of to involuntary childlessness [17].

Variations in fertility knowledge are strongly influenced by social background, personal and family history, as well as educational level [22]. This underscores that the differences between medical and non-medical students found in this study may also be due to factors not assessed such as those mentioned above. In addition, individual personality and the nature of the Academic study may also be responsible for the differences seen in this study.

Our data point to a family planning dilemma among female medical students. 81% of female medical students plan to have children at some point in the future. This strong desire for children was underscored by the fact that the option of abstaining from having children was chosen by no female medical student (0%), whereas female non-medical students chose this option in 5%. Despite this strong will to establish a family, female medical students wanted to have fewer children at a later stage in life compared to non-medical students. Specifically, 66% of female medical students planned to have 2 children compared to 42% of female non-medical students, whereas 3 children were an option for only 13% of female medical students compared to 26% of female non-medical students. Also, most female medical students, ie 60%, did not judge a delivery <29 years as a realistic option in their future plans. These data demonstrate that female medical students seem to strongly anticipate a conflict between a career in medicine and a fulfillment of their family plans. This underscores the need for more support to harmonize female medical careers and family needs.

## Conclusions

In summary, we found that there are marked differences among medical and non-medical students regarding fertility awareness. These data may be helpful to address knowledge gaps among young non-medical Academics. Also, it is reassuring that female as well as male medical students have healthier lifestyles and a more profound interest in and knowledge of fertility issues. This makes them good ambassadors for healthy living and fertility awareness. Lastly, more support for female medical Academics is strongly needed in order to allow them to harmonize their careers and families.

## Electronic supplementary material

Additional file 1: **Fertility awareness questionnaire.** Fertility awareness questionnaire with 5 sections and 43 questions regarding personal data (8 questions), lifestyle factors (9 questions), plans on having children (5 questions), infertility (5 questions), lifestyle and fertility (16 questions). (RTF 11 KB)
