# Supplementary material for: Fertility awareness among medical and non-medical students: a case-control study
Source: Reprod Biol Endocrinol. 2014 Sep 26;12:94. doi: 10.1186/1477-7827-12-94 (PMC4182859; doi:10.1186/1477-7827-12-94)
Supplement: Supplementary file 1 — Additional file 1: Fertility awareness questionnaire. Fertility awareness questionnaire with 5 sections and 43 questions regarding personal data (8 questions), lifestyle factors (9 questions), plans on having children (5 questions), infertility (5 questions), lifestyle and fertility (16 questions). (RTF 11 KB) [file 12958_2014_1264_MOESM1_ESM.rtf]

Additional file 1
Fertility awareness questionnaire. 

Fertility awareness questionnaire with 5 sections and 43 questions regarding personal data (8 questions), lifestyle factors (9 questions), plans on having children (5 questions), age and fertility (5 questions), and lifestyle and fertility (16 questions).

Personal Data

1. How old are you?   _____  years

2. Sex:		□ male
□ female

3. Relationship status:		□ single
				□ in a relationship
				□ married
				□ divorced

4. Do you have a child/children?
□ yes 		□ no

5. What is your field of study?	□ Medicine
					□ Other: ___________________

6. In which term of your studies are you at the moment?
□ 1st-2nd term  	□ 3rd-4th term  	□ 5th-6th term  	□ >6th term

7. How tall are you?
_____ cm

8. How much do you weigh?
_____ kg

Lifestyle Factors

1. Do you consume caffeinated beverages (coffee, black/green/white tea, hot chocolate, cola, energy drinks) on a regular basis?
□ yes	□ no

2. If yes, how many cups/glasses/cans of coffee/tea/hot chocolate/cola/energy drinks do you drink on average per week?
Coffee (1 cup = 200 ml): ______ cups
Black /green/white tea (1 cup = 200 ml): ________ cups
Hot chocolate (1 cup = 200 ml): _________ cups
Cola (1 glass = 250 ml): __________ glasses
Energy drinks (1 can = 250 ml): ___________ cans

3. Do you drink alcohol?
□ yes	□ no

4. If yes, how many glasses of beer/wine/spirits do you drink on average per week?
Wine (1 glass = 0,25 l): ________ glasses
Beer (1 glass = 0,5 l): ________ glasses
Spirits (Cocktails, etc.) (1 glass = 0,02 l): _________ glasses

5. Do you smoke?
□ yes	□ no

6. If yes, how many cigarettes do you smoke on average per day?
□ < 10 cigarettes/day	□ > 10 cigarettes/day

7. Do you exercise?
□ yes, on a regular basis	□ yes, on a non-regular basis	□ no

8. If you exercise on a regular basis, how many hours do you practice on average per week?
_____ hours/week

9. Do you attach importance to a healthy and balanced diet? 
□ yes		□ no		□ sometimes

Plans on Having Children

1. Do you want to have children (have more children) at some point in your life?
□ yes	□ no	□ don't know

2. If yes, how many children would you like to have? 
□ 1	□ 2	□ 3	□ 4	□ 5	□ > 5	     □ don't know  

3. At which age would you like to have your first child? 
□ < 20 years		□ 20-24 years	□ 25-29 years	□ 30-34 years             ⁮  □ 35-39 years	□ 40-44 years	□ ≥ 45 years		□ don't know  

4. At which age would you like to have your last child? 
□ < 20 years		□ 20-24 years	□ 25-29 years	□ 30-34 years             ⁮  □ 35-39 years	□ 40-44 years	□ ≥ 45 years		□ don't know  

5. In case of infertility, which one of the following options would you most likely choose?
□ Assisted reproduction    
□ Adoption 
□ Abstain from having children

Age and Fertility

1. At what age are women most fertile?
□ < 20 years 	   □ 20-24 years    □ 25-29 years     □ 30-34 years    □ 35-39 years	
□ 40-44 years   □ 45-49 years     □ 50-54 years   ⁮□ ≥ 55 years 

2. At what age is the first decrease in a woman's ability to become pregnant?
□ < 20 years 	   □ 20-24 years    □ 25-29 years     □ 30-34 years    □ 35-39 years	
□ 40-44 years   □ 45-49 years     □ 50-54 years   ⁮□ ≥ 55 years

3. At what age is the second marked decrease in a woman's ability to become pregnant?
□ < 20 years 	   □ 20-24 years    □ 25-29 years     □ 30-34 years    □ 35-39 years	
□ 40-44 years   □ 45-49 years     □ 50-54 years   ⁮□ ≥ 55 years 

4. At what age is the first decrease in male fertility?
□ < 25 years	    □ 25-29 years  □ 30-34 years 	   □ 35-39 years    □ 40-44 years
□ 45-49 years   □ 50-54 years  ⁮□ 55-59 years   □ 60-64 years   □ ≥ 65 years

5. At what age is the second decrease in male fertility?
□ < 25 years	    □ 25-29 years  □ 30-34 years 	   □ 35-39 years    □ 40-44 years
□ 45-49 years   □ 50-54 years  ⁮□ 55-59 years   □ 60-64 years   □ ≥ 65 years

Lifestyle and Fertility

1. In my opinion, the consumption of caffeinated beverages affects female fertility in the following way: 
□ The consumption of caffeinated beverages increases female fertility
□ The consumption of caffeinated beverages doesn't affect female fertility
□ The consumption of caffeinated beverages on a regular basis reduces female fertility, occasional consumption of caffeinated beverages doesn't affect female fertility 
□ The consumption of caffeinated beverages reduces female fertility 

2. In my opinion, the consumption of caffeinated beverages affects male fertility in the following way: 
□ The consumption of caffeinated beverages increases male fertility
□ The consumption of caffeinated beverages doesn't affect male fertility
□ The consumption of caffeinated beverages on a regular basis reduces male fertility, occasional consumption of caffeinated beverages doesn't affect male fertility 
□ The consumption of caffeinated beverages reduces male fertility 

3. In my opinion, alcohol consumption affects female fertility in the following way: 
□ Alcohol consumption increases female fertility
□ Alcohol consumption doesn't affect female fertility
□ Alcohol consumption on a regular basis reduces female fertility, occasional alcohol consumption doesn't affect female fertility 
□ Alcohol consumption reduces female fertility   

4. In my opinion, alcohol consumption affects male fertility in the following way: 
□ Alcohol consumption increases male fertility
□ Alcohol consumption doesn't affect male fertility
□ Alcohol consumption on a regular basis reduces male fertility, occasional alcohol consumption doesn't affect male fertility 
□ Alcohol consumption reduces male fertility   

5. In my opinion, smoking affects female fertility in the following way: 
□ Smoking increases female fertility
□ Smoking doesn't affect female fertility
□ Heavy smoking reduces female fertility, occasional smoking doesn't affect female fertility 
□ Smoking reduces female fertility   

6. In my opinion, smoking affects male fertility in the following way: 
□ Smoking increases male fertility
□ Smoking doesn't affect male fertility
□ Heavy smoking reduces male fertility, occasional smoking doesn't affect male fertility 
□ Smoking reduces male fertility   

7. In my opinion, moderate exercise affects female fertility in the following way: 
□ Moderate exercise increases female fertility
□ Moderate exercise doesn't affect female fertility 
□ Moderate exercise reduces female fertility

8. In my opinion, intense exercise affects female fertility in the following way: 
□ Intense exercise increases female fertility
□ Intense exercise doesn't affect female fertility 
□ Intense exercise reduces female fertility

9. In my opinion, moderate exercise affects male fertility in the following way: 
□ Moderate exercise increases male fertility
□ Moderate exercise doesn't affect male fertility 
□ Moderate exercise reduces male fertility

10. In my opinion, intense exercise affects male fertility in the following way: 
□ Intense exercise increases male fertility
□ Intense exercise doesn't affect male fertility 
□ Intense exercise reduces male fertility

11. In my opinion, excess weight affects female fertility in the following way: 
□ Excess weight increases female fertility
□ Excess weight doesn't affect female fertility 
□ Excess weight reduces female fertility

12. In my opinion, underweight affects female fertility in the following way: 
□ Underweight increases female fertility
□ Underweight doesn't affect female fertility 
□ Underweight reduces female fertility

13. In my opinion, excess weight affects male fertility in the following way: 
□ Excess weight increases male fertility
□ Excess weight doesn't affect male fertility 
□ Excess weight reduces male fertility

14. In my opinion, underweight affects male fertility in the following way: 
□ Underweight increases male fertility
□ Underweight doesn't affect male fertility 
□ Underweight reduces male fertility

15. In my opinion, a healthy, balanced diet affects female fertility in the following way: 
□ A healthy, balanced diet increases female fertility
□ A healthy, balanced diet doesn't affect female fertility 
□ A healthy, balanced diet reduces female fertility

16. In my opinion, a healthy, balanced diet affects male fertility in the following way: 
□ A healthy, balanced diet increases male fertility
□ A healthy, balanced diet doesn't affect male fertility 
□ A healthy, balanced diet reduces male fertility
